# Hospital and Institutional News

**Published:** 1918-12-07

**Authors:** 


### HOSPITAL AND INSTITUTIONAL NEWS.

### DR. CLEMENCEAU.

M. Georges Clemenceau, Britain's honoured guest, will no doubt recall his visit to England more than fifty years ago. He qualified as a doctor of medicine in 1865, his thesis being entitled, *De la génération des éléments anatomiques*. Before going to the United States in 1865, whence he returned in 1869, he spent some months in England. In the United States, however, he became a Professor of French literature at a young ladies' seminary at Stamford, a pretty village in the environs of New York, whence he returned to set up in Paris in 1869 as a medical practitioner. But a series of momentous events drew him actively into politics. "M. Clemenceau," a correspondent remarks, "could wield a sword as well as a lancet, and more than one duellist fought shy of him. His cartel, for example, was refused by that turbulent Bonapartist, M. Paul de Cassagnac, although the latter had previously engaged in some dozen duels. M. Clemenceau publicly slapped his face in the Arena, but the other declined to encounter him. M. Clemenceau's swordsmanship was well known at the time."

### A NOTEWORTHY APPEAL.

A big display advertisement has been inserted in the Glasgow newspapers on behalf of the Royal Infirmary, from the pen, we presume, of Mr. R. Morrison Smith, the secretary. In six packed paragraphs, it points out that, unlike military institutions, voluntary hospitals cannot be closed, and that "though peace were declared to-morrow" the work of the Glasgow Royal Infirmary would be likely to increase. The appeal remarks that the total number of patients treated annually are "the numerical equivalent of nearly two Divisions of the British Army, and that the cost of everything needed, except the love, labour, and self-sacrifice of the staff," has nearly doubled. The appeal is made particularly to the generous middle class; which is reminded that after the visits of various working-class deputations to the hospital, "100 additional associations of workers have become subscribers." Yet, the appeal significantly adds, "with all the goodwill in the world the working classes could not of themselves keep up the Glasgow hospitals." The whole concludes with the sum needed. Chairmen and treasurers have not often put their signatures to a better appeal, and the few points we have reproduced should be suggestive to other hospitals.

### THE SUNK HOSPITAL SHIPS: A WHITE PAPER ISSUED.

An historic White Paper has been issued recording the torpedoing of four British hospital ships by German submarines, together with the sworn evidence, in the form of a circular addressed to His Majesty's Diplomatic representatives in Allied and neutral countries. The vessels were the *Rewa*, the *Glenart Castle*, the *Guildford Castle*, and the *Llandovery Castle*. The dispatch, which has been drafted by the Right Hon. A. J. Balfour, says, in reference to the destruction of the *Llandovery Castle* last June, that there can in the circumstances be little doubt that the German commander attempted to slaughter all the witnesses of his crime—to sink the ship without leaving any trace, according to Count Luxburg's notorious phrase. This is the fourth case of deliberate attack without warning on British hospital ships which has been perpetrated by enemy submarines since the commencement of the present year alone (1918). The first three cases have so far been denied by the German Government, who prefers to disbelieve the facts. On the present occasion no denial is possible, since the submarine was actually seen and communicated with by the survivors. "These outrages," Mr. Balfour concludes, "have all been committed in waters in which, by solemn assurance given in writing to the Spanish Embassy in Berlin on October 17 last, and emphatically repeated since that date, the German Government had undertaken that hospital ships should be protected in conformity with the Hague Convention."

### VENICE, ANTWERP, BRUGES—AND CARDIFF?

On November 21, 1918, the date on which the German fleet surrendered, "the light cruiser *Cardiff* was ordered to lead the way, and, if the German ships were out of reckoning, to guide them to the appointed rendezvous." This order of the British Admiral has identified the proudest date in the history of the Navy with the city of Cardiff, and it is hoped to connect it with the war memorial of the city. Since that centres in King Edward VII.'s Hospital, Colonel Bruce Vaughan has happily suggested that one or more of the large wards now being built at a cost of £10,000 should be finished at the charge of the shipowners of the port, and further, that the walls of each such ward should be embellished with the pictures showing "the light cruiser *Cardiff*" conducting the German Fleet to its place of surrender. It will be remembered that the hospital has already wards decorated with historical pictures in encaustic tiles, and that the King admired them on his visit there in 1912. Since the wards would probably be used for women, many of whom will be the wives of men of the Fleet or of the Mercantile Marine, the plan is worthy of the port of Cardiff, and serve to remind the public of the heroism of our seamen and of the generosity of the shipping magnates, who are credited with large profits since the war. There is not necessarily a crime in making a big profit; the crime consists in not spending it magnificently. If the merchants were proud to adorn the walls of public buildings in Venice, in Antwerp, and in Bruges, the Cardiff shipowners will hardly wish their own port to take a back seat now the chance has come.

### PADDINGTON GREEN CHILDREN'S HOSPITAL.

In order to obtain the sum annually required for the maintenance of a cot in the Paddington Green Children's Hospital by the parish of St. Stephen's,

Westbourne Park, Mr. E. P. Woollcombe is giving an entertainment in St. Stephen's Schools on Saturday, December 7. £35 is required this year, and it is hoped that by means of this entertainment and special subscriptions the whole amount may be forthcoming. It is certain the members of St. Stephen's congregation, who have maintained the cot for many years, will do their best to achieve this result. Already some £15 has been received in subscriptions.

### THE WELFARE OF THE WORKER.

The Institute of Hygiene has decided to make an Industrial Council, representative of capital, labour, and medicine, a special section of its General Council. The object of this Industrial Council will be to investigate the diseases and disadvantages of industrial life and to prevent them by promoting and spreading a better knowledge of hygienic measures. It is considered by the Medical Council of the Institute that not only good housing and surroundings are required, but that proper foods and feeding are of equal, if not primary importance, and that recreation (mental hygiene) also deserves more consideration to maintain the health and fitness and the contentment of the worker.

### THANK-OFFERINGS FOR PEACE ALREADY RECEIVED.

The Royal Victoria and West Hants Hospital, Bournemouth, in common with other institutions, has received a considerable sum as a peace thank-offering to mark the signing of the Armistice. The donors include two life governors of the hospital, Mr. J. E. Cooper-Dean, J.P., who has given three hundred guineas to name a bed at each branch of the institution, and Mr. C. Hodges, J.P., who has given one hundred guineas to name a bed at the Boscombe branch. Mrs. Cassel, who for a long time has taken an active interest in the welfare of the hospital, has given £100 "in honour of the first news of Peace" and she has now been made a life governor. The voluntary hospitals have done magnificent work during the war, and it is appropriate that they should be prominent in the minds of our countrymen at this time of national thanksgiving. On the signing of the Peace Treaty many others are sure to follow the example already set.

### A LIFT FOR BIRMINGHAM CHARITIES.

Subject to certain life interests a large share of the estate of the late Mr. Julius Jones will go to the Queen's Hospital, the Blind Institution, and the Birmingham and Midland Hospital for Nervous Diseases. The first two charities are well known, but the other not so. This provincial special hospital has not afforded much information to the usual books of reference as to the extent of its work, and whether it has connected with it any of the better known neurologists of the Midlands, such as Dr. Stanley Barnes and others, we have not at the moment any means of ascertaining. But Mr. Jones's generosity may, at some future date, afford a nucleus for a fund for the provision of a useful centre for the study of the diseases of the nervous system to be established or developed in Birmingham. Hospitals will undoubtedly become more and more consultative in the next few years, and this condition will very probably be hastened and encouraged by a Ministry of Health. Mr. Jones has left the residue of his estate to a number of midland hospitals, and has included St. Dunstan's Hostel for the Blind.

### INFIRMARIES' STAFFS AND INFLUENZA.

The serious difficulties which the medical and nursing staffs in infirmaries have had to surmount owing to the influenza epidemic are shown in a report presented to the Lambeth Board of Guardians by Dr. Lionel Baly, medical superintendent at their infirmary. Unfortunately, Dr. John McCaw, whose death we recorded recently, was a victim to the complaint, whilst in addition three other members of the medical staff and twenty-one nurses contracted influenza. As a result the wards were understaffed and the patients did not receive sufficient attention. The number of patients admitted rose rapidly, over 100 being sent to the infirmary in three days. Three small wards, which had been closed during the summer, could not be opened owing to lack of staff, so that many wards were overcrowded. During a fortnight Dr. Baly had refrained from admitting patients suffering from influenza from the inner part of the borough other than those who presented themselves at the infirmary and were too ill to be sent home. This entailed considerable addition to the district work, but up to the present none of the cases treated at home had died, and several had recovered, in spite of the fact that they had developed pneumonia. He had come to the conclusion that there was considerable risk in moving patients from their homes to the infirmary after they had developed signs of pneumonia.

### A DOCTOR'S DILEMMA.

At Wolverhampton a private in the South Staffords has died because apparently he could gain no medical attendance in consequence of the Army Order which forbids a civilian practitioner to attend a soldier. The man, according to evidence at the inquest, feeling ill, called Dr. Grant, who, having no legal authority to prescribe for him, sent him to the Drill Hall and advised hospital treatment. From the hall Hill went to the military hospital at Lichfield, where there was no room. He returned to Wolverhampton, telegraphed to his depôt, and was ordered to go to the hospital from which he had been already turned away. Thereupon he applied for admission to Wolverhampton General Hospital, but apparently there was no means for transferring him there. A local doctor who was sent for was himself ill, and Dr. Grant arrived in time, so to speak, to sign Hill's death certificate. In criticising the regulation which forbade him to prescribe for a soldier, Dr. Grant was within his rights; but in contending that all doctors should have authority to attend soldiers he overlooked the reason for a rule which, generally speaking, is not far to seek. All rules, however, are occasionally honoured in the breach. This was such an occasion; and if Private Hill was ill enough to break the regulations by applying to Dr. Grant, could not Dr. Grant have said to him in the first instance, "If you prove to me that you are unable to gain prompt admittance to a military hospital, in view of the urgency of your case, I will prescribe for you"?

### ARMY COUNCIL INSTRUCTION 1293.

Under this head lectures and general classes for convalescent officers and men to include those in hospital are to be extended. Central hospitals, those with more than a fixed number of beds, will have to add to their staff an education officer. His duties will include not only the provision of classes and lectures for the men in the hospital to which he is attached, but where possible to extend their benefits to the auxiliary hospitals of the district. Under the commandant the education officer will be responsible for general education, occupational training, and technical instruction. The British Red Cross Society has placed certain funds at the disposal of the War Office to extend this work. Where workshops have already been formed under the Ministry of Pensions in the neighbourhood of a central hospital, the education officer will co-operate with the local representative of the Ministry.

### RELIEVING OFFICER'S DILEMMA.

At a recent meeting of the Southwark Board of Guardians it was reported that Mr. F. G. Carlisle, one of their relieving officers, had to be admitted to the Newington institution as a patient seriously ill with influenza. His wife and five children were all at home at the same time suffering from the same complaint, and it was stated that the only possibility of saving him was to admit him to the institution. In order to get over the financial difficulty the master of the institution (Mr. W. J. Swinneck) agreed to accept Mr. Carlisle as his guest during his stay there, and the Guardians thanked the master for his consideration. The officer, thanks to the institutional treatment he received, is now convalescent.

### CAPTAIN L. HADEN GUEST, R.A.M.C., AND PARLIAMENTARY HONOURS.

Captain L. Haden Guest, M.C., of the R.A.M.C., is the Labour Parliamentary candidate in Central Southwark (West Newington). Captain Haden Guest in pre-war days was an assistant medical inspector of secondary schools under the London County Council, and medical superintendent of St. George's Dispensary and School Clinic in the Borough. He had served in the R.A.M.C. during the Boer War, and, consequently, when the war broke out he at once took service again. He organised hospitals under the French Red Cross, and was one of the founders of the Anglo-French Committee of the British Red Cross. Since 1914 he has served in France and Palestine, and came home after the Armistice to take up the political fight in Central Southwark. Captain Haden Guest was awarded the Military Cross for rescuing wounded under fire during the advance of the 58th Division at Paschendaele Ridge. He afterwards took part in the advance of General Allenby in Palestine. The health and feeding of children has been his special care, and long before the war the facts of life in this borough had convinced him of the need of drastic improvement of the conditions of the lives of the people by Parliamentary action.

### AN HISTORIC HOSPITAL SERVICE.

The Derbyshire Royal Infirmary is claimed by one of its friends to have been the first hospital to hold a thanksgiving service, and further that the first of all such services was that held in the chapel of the Royal Infirmary. The hospital board, we understand, was in session on Monday November 11, and the board decided immediately to hold a thanksgiving service, which was held at 12.30 P.M., that is to say within an hour and a half of the last shot. The Rev. A. T. Humphreys, the hospital chaplain, conducted the service, and the lesson and the 46th Psalm were read by the Rev. F. C. Player. The chapel was packed, and the congregation included Mr. A. Cox, the president, the medical staff, the secretary, Mr. Forster, the matron, Miss Sutcliffe, the sisters and the nurses, and the servants. Such patients as could attend were present, including a bevy of convalescent soldiers. Some interesting details have been happily preserved by a writer in the *Derby Reporter*, who, as one "privileged to be present at the service, desires to put the occasion on record . . ." His impression may be given in his own words: "The service, though short, was in every way memorable both by reason of its spontaneity and unanimity, illustrative of the spirit of religion which permeates the work of the infirmary, where surely, if anywhere in the town, the influence of our Great Healer actuates the willing service so constantly and ungrudgingly rendered."

### THE PREVENTION OF TETANUS.

According to Louis Bazy, who contributes an article on the subject to the October "French Supplement to the *Lancet*," the experiences of the war have revolutionised the prevention of tetanus with marked success. The principles on which the new departure are based are the combating of the immediate danger by the subcutaneous injection of antititoxin, and of the permanent menace by antitetanic vaccination. The immunity conferred by antitoxin is limited to fifteen days, and as postseric tetanus may occur up to 212 days it is obvious that a more durable protection is indicated. The emulsion of bacilli employed is lethal to a guinea-pig of 400 grammes, in a strength of 1 in 10,000; and this is diluted with a third of iodised solution (1 in 200). The injections are given in consecutive doses of 4, 8, 12 c.cms., neither local nor general phenomena being produced. Let us hope that the opportunity for the trial of this discovery in the armies of ourselves and of our Allies may be indefinitely postponed, but for this prospect of the more certain prevention of one of the most heartrending accidents incidental to war wounds we are duly grateful to our French scientific comrades.

### GLYCERINE WITH NO RESTRICTIONS.

In the interval between the writing and publication of the note regarding glycerine (see The Hospital, November 30), the Ministry of Munitions have now intimated that glycerine may be used for any purpose desired, instead of "strictly medical purposes." Although no large quantities are yet obtainable from the wholesale druggists, it is anticipated that hospitals will soon receive fairly adequate supplies. It is likely, too, that the best quality of castor oil will shortly be again obtainable and, whilst the "neutralised seconds" has proved fairly satisfactory for general use, children's hospitals have found it not so good for making up the familiar emulsions of castor oil, used so largely in the treatment of infants and young children.

### PNEUMONIC WARDS AT BURY.

In order to meet the needs of the civilian population, now in the throes of the influenza-pneumonia epidemic at Bury St. Edmunds, the West Suffolk General Hospital has transferred its military patients to auxiliary hospitals in all cases where it was found possible to move them, and has placed the two wards formerly occupied by them at the disposal of the medical men in the borough for patients suffering from either of these diseases. The only letter of admission required is one from the doctor attending the case, certifying that he considers hospital treatment advisable. By doing this the hospital is able to receive forty-four patients who would otherwise have had little chance of proper nursing in their own homes. At Bury, as in other districts, whole families are sometimes attacked. A word of praise is due to the nursing staff, whose members, already feeling the strain of the last four years, during which they have been often very short-handed, are now tackling the epidemic with the help of workers from the Voluntary Aid Detachments.

### PAYMENT FOR LYING-IN BEDS.

In connection with the Maternity and Child Welfare Scheme the Health Committee of the Southampton County Council has arranged with the authorities of the Royal Hampshire County Hospital at Winchester for the reservation of beds for confinements in difficult cases upon the following terms.

(1) The hospital will receive maternity cases which require special care, or which should receive hospital treatment owing to unsatisfactory home conditions. (2) The County Council will pay the cost of adapting the "Special" Nightingale Ward and the Sister's Room adjoining for a maternity ward and labour room, and also for the provision elsewhere for a room for the sister (cost about £400). (3) The County Council will pay a sum of £2 2s. per case, and will also pay 6s. per day per occupied bed, with a minimum payment of £200 per annum. (4) The County Council will find or pay for drugs and dressings. (5) The hospital will provide beds, cots, instruments, and apparatus, but the County Council must make good breakages and wear and tear. (6) The hospital authorities propose at their own expense to send one of their staff sisters to the Rotunda Hospital, Dublin, for a four months' course. (7) This agreement to be from year to year, with six months' notice on either side to terminate, but should the hospital determine the agreement before the end of the sixth year, the hospital will refund one-sixth of the sum expended on the adaptation of the ward, etc., for each year unexpired.

### INFLUENZA AND BURIALS.

One of the most serious difficulties which arose from the influenza epidemic was the burial of those who succumbed. At Norwood Cemetery, for instance, the interments in one fortnight numbered 243, as against sixty in the same period twelve months ago, whilst as many as thirty-eight bodies were lying in one week in the mortuary of the Southwark Board of Guardians at their Newington institution awaiting removal to the homes of their relatives. The bodies of some babies, it is stated, had to remain fourteen days before they could get interred. One difficulty was lack of hearses, and the Guardians had to call in whatever help they could, in many cases the bodies being conveyed to the homes in the only available vehicle—a fish-cart. In the East End of London the same unsatisfactory state of affairs existed, and efforts have been made to induce the Government to come to the assistance of the civil population. Here, surely, was a clear case for the liberation of men, either coffin-makers or undertakers, who were serving in the Home Forces, but the responsible officials did not release the men. One undertaker in South London has three sons in the Army, all serving in units in the London area, but he has been unable to secure their services, and has been left alone with twenty-six horses to look after. These men should have been released, at least for a time, in order that the state of affairs in regard to burials in London could have been relieved.

### THIS WEEK'S DRUG MARKET.

The general "easier" tendency in the prices of drugs continues, and buyers, in the hope that before long the downward movement will become quicker, are buying very cautiously. Sugar of milk is again lower; this is one of the few articles that have "slumped," the price within a short period having been cut nearly in half. Chloral hydrate moves steadily downwards, and the same may be said of various other drugs, as, for instance, benzoic acid, gentian root, atropine, guaiacol carbonate, senega root, bromides, cream of tartar, lanoline, saccharin, phenolphthalein, soft paraffin, saffron, liquorice juice, and terpineol. Glycerine is available at prices which can be considered reasonable in view of the circumstances, but as supplies become more plentiful we may expect to see values recede. Better qualities of medicinal castor oil should shortly be available in increasing quantities.

